# Parenting motives: Validation of the Italian version of the parental care and tenderness scale

**DOI:** 10.3389/fpsyg.2022.1064626

**Published:** 2023-01-16

**Authors:** Luigi Castelli, Tania Garau, Luciana Carraro

**Affiliations:** Department of Developmental and Social Psychology, University of Padova, Padua, Italy

**Keywords:** parenting motives, individual differences, validation, spontaneous behaviors, political orientation

## Abstract

Relevant individual differences can be observed in relation to parenting motives. The Parental Care and Tenderness (PCAT) scale is an important tool aimed at assessing them. We here investigated the psychometric properties of an Italian version of the scale (*N* = 946). The scale had a very high reliability and its internal structure closely reproduced the one obtained in different cultural contexts. Two major subscales, namely nurturance and protection, could be identified. In addition, we explored the validity of the scale in relation to a novel domain. Individual differences in parenting motivations, and more specifically those related to nurturance, emerged to be associated with a spontaneous behavioral tendency to approach children. Scores in the PCAT, and more specifically scores in the protection subscale, were also associated with a more conservative political orientation. The present work contributes to the growing literature about the key role of parenting motives in affecting social behaviors.

## Introduction

Since the publication of Maslow’s influential theory Maslow (1943), the presence of multiple, independent, and hierarchically-structured motivational systems has been postulated. Recently, the original pyramid of needs proposed by Maslow has been enriched including a more nuanced differentiation between various needs (Kenrick et al., 2010). In particular, the care for children and the so-called parenting motivation have been placed at the top of the pyramid. From an evolutionary perspective, not only reproductive success, but also the maximization of the survival rate of the offspring is fundamental, especially for species – like the human species – that are characterized by a long gestation period and numerically limited offspring that require several years before achieving autonomy. Hence, psychological mechanisms aimed at supporting the care and protection of children would be highly adaptive (Schaller, 2018, 2020). In line with the early proposals of Lorenz (1943), it has indeed been demonstrated that the mere presence of infant facial features, as well as children-related sounds and smells, may activate specific patterns of behavioral responses and neural activity (Kringelbach et al., 2016), which in turn support caregiving.

Although the activation of a motivational system aimed at supporting the care and protection of children is more directly relevant in the case of parents (Thompson-Booth et al., 2014), faces of babies attract the attention and appear to be more rewarding, as compared to adult faces, for both parents and non-parents (Brosch et al., 2007; Parsons et al., 2011; Senese et al., 2013). Gender differences have also been observed. Because women are more strongly involved in the care of children due to both biological (e.g., breast feeding) and socio-cultural factors (i.e., cultural norms forcing women to assume the role of primary caregivers), they often tend to exhibit higher sensitivity to children (Buckels et al., 2015) as compared to men. In addition, a motivational system related to the care of children may be differently active in different periods of one’s own life (e.g., during the transition to parenthood) and as a function of the presence of contextual factors that can trigger such a motivational system.

Much research has focused on experimental paradigms in order to investigate the affective, cognitive, and behavioral consequences of the temporary activation of the motivational system aimed at assisting children. Typical manipulations include asking respondents to focus on their actual (or potential) parental role, presenting photographs of cute children or making salient their vulnerability. Findings indicate that, under such conditions, people become more risk-averse and less trustful toward strangers (Eibach and Mock, 2011), more prejudiced towards threatening ethnic outgroups (Gilead and Liberman, 2014), harsher in their judgment of norm violation (Eibach et al., 2009), and with a decreased short-term mating orientation (Beall and Schaller, 2019).

Besides situational variations in the salience of parental care motivations, chronic individual differences in relation to the activation of the parental care motivational system exist which go beyond the overall distinction between parents and non-parents (or women and men). In order to assess such individual differences, a questionnaire measure has been recently developed. The scale originally proposed by Buckels et al. (2015) - called *Parental Care and Tenderness* (PCAT) questionnaire - comprises 25 items with 5 underlying factors. The reliability of the whole scale emerged to be very high (Cronbach’s *α* = 0.95), as well as the reliability of the 5 subscales each including 5 items (Cronbach’s αs > 0.85; Buckels et al., 2015). The five factors tap distinct albeit highly intercorrelated dimensions. In particular, one factor is related to the predisposition to protect children from harm. The other four factors are related to the liking for children, the strength of caring responses, the experienced tenderness triggered by children engaging in cute behaviors or in a condition of distress. In addition to a very high internal consistency, the scale shows good validity. First, the scores obtained from the PCAT significantly differ for known groups (Cronbach and Meehl, 1955). Higher scores were observed in the case of parents as compared to non-parents, and for female as compared to male respondents. Construct validity was also examined by assessing the correlation with the responses to other conceptually related measures, such as the Nurturance subscale of the Personality Rating Form (Jackson, 1967). The properties of the PCAT have been also analyzed in relation to its predictive validity. Scores on the PCAT were associated with the affective reactions aroused when exposed to images of both distressed and nondistressed babies, and this was true even when controlling for individual differences in empathic concern (Buckels et al., 2015). In addition, a significant association was found with the reward value of infant faces. Participants in one specific study (Buckels et al., 2015, Study 5) were allowed to perform behaviors aimed at either prolonging or reducing the exposure time to pictures of babies and adults. As hypothesized, higher scores in the PCAT were predictive of longer times spent viewing baby, but not adult, pictures. Responses on the PCAT were also predictive of reactions to infants even after controlling other relevant constructs (e.g., personality traits, behavioral activation and inhibition, emotionality), thus supporting the good discriminant validity of the scale.

One key goal of the present work is to validate the PCAT in a different cultural context, namely in Italy. As a first step, the factorial structure of the scale will be assessed following the approach employed in the original study (Buckels et al., 2015). A step further, whereas Buckels et al. (2015) mainly focused on the overall score derived from the responses to all 25 items, Hofer et al. (2018) have more recently proposed that two main subscales can be identified: Nurturance and Protection. Hence, we attempted to provide further evidence about the empirical solidity and theoretical relevance of this proposal (Hofer et al., 2018). Critically, some relevant outcomes, such as the time spent looking at cute babies, appear to be better predicted by nurturance, whereas other variables, such as restrictive parenting practices, are better predicted by protection. At a more general level, nurturance appears to be more strictly interconnected with approach-oriented responses towards children (Hofer et al., 2018) and this idea is also supported by neuropsychological evidence showing that self-reported maternal nurturance (but not protection) is correlated with the activation of reward-associated brain regions when exposed to infant faces (Endendijk et al., 2020). Because of the importance of approach-oriented responses, we extended the validation of the PCAT scale by administering to a subsample of participants a computerized task aimed at assessing spontaneous approach-avoidance tendencies (i.e., the Visual Approach/Avoidance by the Self Task; VAAST; Rougier et al., 2018; Aubé et al., 2019). We hypothesized that respondents scoring higher on the PCAT would also be comparatively faster to approach pictures of infants rather than pictures of adults. More specifically, we will also test whether responses to items related to nurturing, as compared to protection, are more strongly associated with the spontaneous behavioral tendencies to approach children.

Although the primary goal of the paper was to validate the Italian version of the PCAT, data provided the opportunity to further explore the correlation between the scores in the PCAT and other relevant individual differences in apparently unrelated domains. As convincingly argued by Kerry and Murray (2018), there are several theoretical reasons to expect that parenting motives may be associated with more conservative attitudes. For instance, parenting-related thoughts are related to increased risk-aversion and sensitivity to threats (Fessler et al., 2014; Gilead and Liberman, 2014), and conservative attitudes have been proven to represent a functional response to perceived threats (e.g., Jost et al., 2003). In addition, social conservativism is associated with a preference for anti-abortion attitudes and more traditional family structures, and, in turn, long-term mating preferences are associated with stronger parenting motivations (Beall and Schaller, 2019). Importantly, previous empirical evidence strongly supports the presence of a strict interconnection between parenting motivations and conservative attitudes (Kerry and Murray, 2018, 2020; Kerry et al., 2022). Relying on this well-established finding, we will test the correlation between these two constructs in a different socio-political context as a further way to assess the predictive validity of the proposed scale and, more specifically, we will explore whether political orientation is more strictly connected to either nurturance or protection, as defined by Hofer et al. (2018).

## Study 1

### Participants

Nine hundred forty-six Italian participants (29.2% males, 70.3% females, 0.5% other; 59.3% parents and 40.7% non-parents) aged between 18 and 83 years (*M* = 32.65; *SD* = 14.63) completed an online questionnaire. Participants were recruited using snowball sampling and the network of contacts of several research assistants. The only constraint was their minimum age (i.e., 18 years). All participants provided an informed consent.

### Procedure

Participants were first administered the Italian translation of the Parental Care and Tenderness Questionnaire (PCAT; Buckels et al., 2015). The translation was carried out by the authors of the present work who agreed on the final formulation of the items (see Table 1). The response scales (i.e., 5-point Likert scales) closely followed those proposed by Buckels et al. (2015). Finally, participants were asked to report their age, gender, and parental status.

**Table 1:** The Italian translation of the 25 items of the PCAT.

	Factor					Comm.
PCAT item	1	2	3	4	5	
Factor 1 (Caring)						
13. I bambini generalmente profumano	**−0.64**	−0.01	−0.10	−0.03	0.10	0.38
01. Quando vedo dei neonati voglio tenerli in braccio	**−0.64**	−0.08	−0.01	0.23	0.03	0.69
10. Le piccole dita delle mani e dei piedi di un bambino sono davvero adorabili	**−0.51**	−0.04	−0.02	0.39	0.01	0.55
03. Quando vedo un bambino tra le braccia di qualcuno, mi si scalda il cuore	**−0.50**	−0.12	−0.07	0.37	0.05	0.65
06. I bambini mi riempiono il cuore	**−0.50**	−0.24	−0.10	0.30	0.02	0.68
Factor 2 (Liking)						
05. Penso che i bambini siano fastidiosi (R)	−0.07	**−0.85**	−0.04	−0.02	−0.03	0.78
02. Quando sento un bambino piangere, il mio primo pensiero è “stai zitto!” (R)	0.01	**−0.84**	−0.05	−0.02	−0.01	0.73
11. Non mi piace avere intorno dei bambini (R)	−0.04	**−0.84**	0.02	0.04	0.01	0.75
08. Non sopporto che i bambini si lamentino sempre (R)	−0.12	**−0.71**	−0.07	−0.11	−0.12	0.53
14. Se potessi, assumerei una tata che si prenda cura dei miei bambini (R)	0.22	**−0.53**	−0.06	0.12	0.14	0.50
Factor 3 (Tenderness-Negative)						
24. Vedi un bambino scivolare e cadere a terra	0.12	0.04	**−0.85**	0.09	−0.02	0.72
23. Vedi un bambino che sta male	0.13	0.05	**−0.84**	−0.01	0.04	0.68
17. Senti un bambino che inciampa e cade iniziando a piangere	0.04	−0.02	**−0.83**	0.10	0.01	0.77
18. Senti un bambino piangere forte su un aereo	−0.21	−0.13	**−0.68**	−0.07	−0.02	0.70
21. Devi cambiare il pannolino sporco di un bambino	−0.25	−0.09	**−0.61**	−0.07	0.02	0.65
Factor 4 (Tenderness-Positive)						
20. Fai ridere un bambino più e più volte facendo facce buffe	−0.10	−0.07	−0.01	**0.74**	0.03	0.56
25. Vedi un padre che, come gioco, lancia in aria il suo bambino che ride	0.24	0.04	−0.06	**0.71**	0.01	0.40
22. Un bambino ti manda dei baci per salutarti	−0.21	−0.01	−0.06	**0.70**	0.01	0.58
19. Guardi un bambino mentre muove i suoi primi passi e cadere delicatamente per terra	−0.16	−0.04	−0.18	**0.66**	−0.01	0.60
16. Un neonato stringe la sua mano intorno al tuo dito	−0.21	−0.07	0.01	**0.68**	0.01	0.51
Factor 5 (Protection)						
07. Farei del male a chiunque rappresentasse una minaccia per un bambino	0.06	0.01	0.01	−0.02	**0.81**	0.69
12. Non mostrerei pietà per chi è stato un pericolo per un bambino	−0.08	0.08	0.01	−0.03	**0.73**	0.57
04. Mi sentirei in dovere di punire chiunque abbia provato a ferire un bambino	0.06	0.02	−0.01	0.08	**0.70**	0.47
15. Userei ogni mezzo necessario per proteggere un bambino, anche se dovessi ferire qualcuno	−0.05	−0.11	−0.03	−0.08	**0.71**	0.48
09. Preferirei andare a letto affamato piuttosto che lasciare un bambino senza cibo	−0.34	0.01	−0.11	0.04	0.30	0.23

The number before each item refers to the numbering of the items provided by Buckels et al. (2015). Extraction method was principal axis factoring with an oblique (Oblimin) rotation. Factor loadings above 0.4 are bolded. Reverse scored items are indicated with an (R). Communalities are reported in the last column (i.e., comm.)

## Results

### Factor structure

After appropriate rescaling (i.e., reverse scoring items on the Liking subscale), an exploratory factor analysis, based on the responses of the whole sample, with oblique rotation (Oblimin) was performed on the 25 items of the PCAT. An oblique rotation was performed because factors were expected to be intercorrelated (see Buckels et al., 2015). The analysis showed one main factor accounting for 32.47% of the variance. Four other minor factors emerged accounting for 9.05, 7.81, 6.78, and 4.23% of the variance, respectively (see Table 1 for factor loadings). The factorial structure of the Italian version closely reproduced the one obtained by Buckels et al. (2015). Only one item (i.e., item 9) that was expected to load on the protection factor presented a factor loading higher for the care than the protection factor. Notably, also in the original validation (Buckels et al., 2015) this item was bifactorial. Table 2 shows the bivariate correlations between the scores related to the five factors and their respective reliability. Descriptive statistics are reported in Table 3. The internal reliability of the PCAT was also high (Cronbach’s *α* = 0.90). Following the proposal by Hofer et al. (2018), we also carried out a confirmatory factor analysis on the 10 items of the PCAT-pn scale in order to assess the goodness of a 2-factor model. Following Hofer et al. (2018), correlated residuals for the three pairs of items in the PCAT-pn Nurturance subscale that loaded on three separate factors of the full 25-item PCAT Scale (Buckels et al., 2015) were included in the CFA. This 2-factor model provided good fit to the data, *χ*^2^(31) = 85.667, *p* < 0.001, Comparative fit index (CFI) 0.991, Root mean square error of approximation (RMSEA) 0.043. The scores on the Nurturance and Protection subscales were moderately correlated, *r* = 0.291, *p* < 0.001.

**Table 2:** Correlation between the 5 factors and internal reliabilities (Cronbach’s alpha within parentheses on the diagonal).

	PCAT factors				
PCAT Factors	Caring	Liking	Protection	Tenderness-positive	Tenderness-negative
Caring	(0.84)				
Liking	0.45	(0.82)			
Protection	0.33	0.18	(0.72)		
tenderness-positive	0.67	0.34	0.28	(0.82)	
Tenderness-negative	0.54	0.35	0.31	0.49	(0.86)

**Table 3:** Means and standard deviations (in parentheses).

	Whole sample (*N* = 946)	Male (*N* = 276)	Female (*N* = 665)	Parents (*N* = 561)	Non-parents (*N* = 385)
PCAT (25-items)	3.77 (0.62)	3.62 (0.63)	3.84 (0.61)	3.86 (0.58)	3.64 (0.65)
Caring factor	3.88 (0.87)	3.54 (0.83)	4.03 (0.83)	3.98 (0.81)	3.73 (0.93)
Linking factor	3.51 (0.97)	3.50 (0.91)	3.52 (0.99)	3.60 (0.94)	3.38 (0.99)
Protection factor	3.93 (0.74)	3.90 (0.74)	3.95(0.74)	3.99 (0.71)	3.85 (0.78)
Tenderness-positive factor	4.37 (0.70)	4.19 (0.76)	4.45 (0.65)	4.44 (0.62)	4.26 (0.78)
Tenderness-negative factor	3.17 (0.1.05)	2.98 (1.03)	3.25 (1.05)	3.30 (1.06)	2.97 (1.00)
Protection subscale	3.80 (0.84)	3.78 (0.85)	3.81 (0.83)	3.84 (0.82)	3.73 (0.87)
Nurturance subscale	3.92 (0.84)	3.65 (0.84)	4.03 (0.81)	4.03 (0.79)	3.76 (0.88)

### Known-groups validity: Male–female and parent–non-parent differences

We first compared the scores of male and female respondents. Female respondents displayed higher scores on the PCAT, *t* (939) = 4.98, *p* < 0.001, *d* = 0.36, and, more specifically, on the two Tenderness factors and on the Care factor (all *p*s < 0.001). No difference emerged on the Liking and Protection factors (*p*s > 0.38). When considering the PCAT-pn (Hofer et al., 2018), female respondents displayed higher scores in relation to the Nurturance, *t* (939) = 6.50, *p* < 0.001, *d* = 0.48, but not Protection (*p* = 0.28) subscale.

As for the comparison between parents and non-parents, parents displayed higher scores on the PCAT, *t* (944) = 5.53, *p* < 0.001, *d* = 0.37, as well as on each of the 5 factors (all *p*s < 0.005). When considering the two factors derived from the PCAT-pn (Hofer et al., 2018), parents displayed higher scores in relation to both the Nurturance, *t* (944) = 4.94, *p* < 0.001, *d* = 0.33, and Protection, *t* (944) = 2.08, *p* = 0.038, *d* = 0.14, subscale.

## Study 2

### Participants, materials, and procedure

A subsample of participants who were recruited in Study 1 (*N* = 174; 33.3% males and 66.7% females; *M*_age_ = 30.66 years; *SD* = 12.46; 18.4% parents) were also involved in Study 2.

### Procedure and materials

Participants performed an additional task, namely the VAAST (Aubé et al., 2019) that was administered *via* the online software PsyToolkit (Stoet, 2017). All participants filled in the PCAT after the VAAST. The VAAST required participants to categorize pictures as either depicting infants or adults. Twenty images depicting White infants and 20 images of White adults were used. In each trial, participants were required to place the index finger of their dominant hand on the H key and press it in order to make the target stimulus appear. The target stimulus appeared on a simulated street background (see Aubé et al., 2019), and, depending on the provided instructions, participants were required to move toward the target and press the Y key or to move away from the target and press the N key. In one block of 30 critical trials, the Y key had to be used for categorizing infants and the N key for categorizing adults. In another block of 30 trials, the key assignment was reversed. The order of these two blocks was counterbalanced across participants. Before each of the two critical blocks, participants completed a training block (10 trials) in which they were provided feedback in case of incorrect responses (i.e., a red X). Importantly, when pressing the Y key (i.e., move toward) the target image on the screen zoomed in and the background image zoomed out, so that the visual feedback gave participants the impression they were actually moving toward the target. In contrast, when pressing the N key (i.e., move away) the target image on the screen zoomed out and the background image zoomed in. Afterwards, participants were instructed to return on the H key and press it for starting the following trial.

### Results

Trials in which incorrect responses were provided were removed (i.e., 2.2%). For each participant, two scores were then computed based on the average time needed to categorize pictures of infants when performing either an approach or avoidance movement. The same was done for pictures of adults. Scores were then submitted to a 2 (target: infant vs. adult) × 2 (movement: approach vs. avoidance) × 2 (gender of the respondent) x 2 (parenthood status: parent vs. non-parent) analysis of variance with the last two factors between-participants. The main effect of the target emerged, *F* (1,170) = 8.002, *p = 0*.005, *η*^2^*_p_* = 0.045, due to faster categorization time for infant as compared to adult pictures. No other significant effect emerged.

Although at the sample level there was no overall tendency to approach children faster than adults, our major focus of interest was related to the analysis of individual differences. To this end, we first computed a summary score based on the responses in the VAAST that captures the different behavioral tendencies towards infants and adults [i.e., (approach_adults + avoidance_children) – (approach_children + avoidance_adults)]. Higher values indicate a stronger tendency to move towards children as compared to adults. This score was then correlated with the summary score on the PCAT. In line with the hypothesis, findings showed a significant, albeit weak, positive correlation, *r* (174) = 0.273, *p* < 0.001.

As a second step, from responses to the PCAT we calculated a score for both the nurturance (6 items) and protection (4 items) subscales following the suggestion by Hofer et al. (2018). These two scores were then entered as predictors in a regression analysis with the summary score in the VAAST as a dependent variable. Findings demonstrated that nurturance was a significant predictor, *β* = 0.225, *p* = 0.005, 95% CI [35.46, 197.04], whereas protection was not related to the behavioral tendencies to approach children vs. adults, *β* = 0.053, *p* = 0.51, 95% CI [−55.46, 111.42].

## Study 3

### Participants and procedure

A subsample of participants who were recruited in Study 1 but not in Study 2 (*N* = 486; 30% males, 69.5% females, 0.4% other; *M*_age_ = 37.19 years, *SD* = 15.62; 278 parents and 208 non-parents) was also asked to report the political orientation. Political orientation was assessed with 3 items requiring participants to report along a continuum ranging from 0 (closer to the left) to 100 (closer to the right) their self-placement as for their overall political ideology, their views about economic issues, and their views about social issues.

### Results

The responses to the 3 items assessing political orientation were strongly intercorrelated (0.74 < *r*s < 0.85; Cronbach’s *α* = 0.92) and an average score was thus computed (*M* = 39.18; *SD* = 25.64). We then conducted a linear regression analysis, with PCAT, parenthood status, gender, and age as independent variables predicting political orientation. Both gender, *β* = −0.125, *p* = 0.006, 95% CI [−11.74, −2.00], and age, *β* = −0.124, *p* = 0.03, 95% CI [0.016, 0.391], significantly predicted political orientation, indicating that male and older respondents displayed a more conservative political orientation. Most importantly, parenthood status was not a significant predictor (*p* > 0.82), whereas higher scores on the PCAT were associated with a more conservative political orientation, *β* = 0.150, *p* = 0.003, 95% CI [2.18, 10.65].

**Figure 1:**
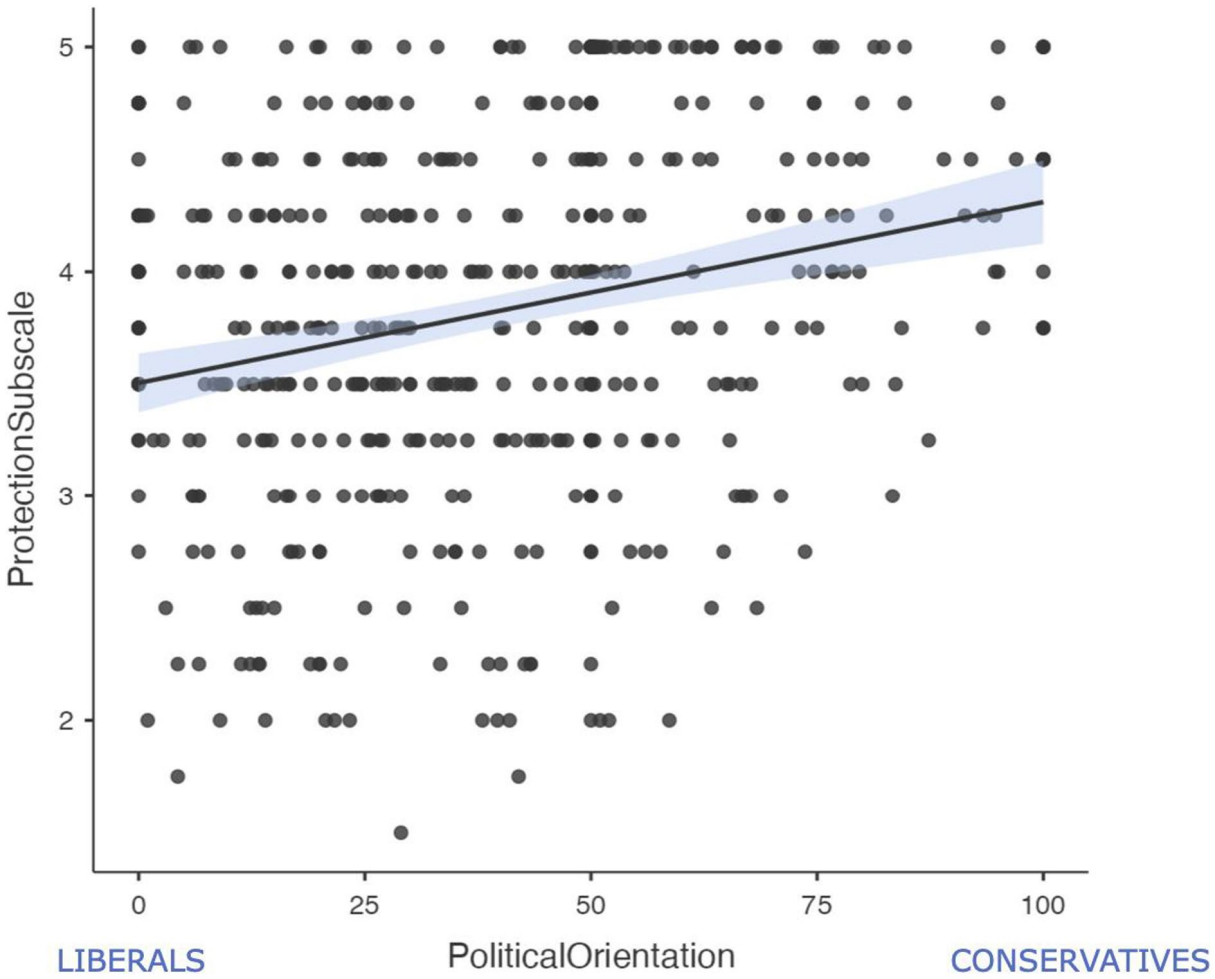
Correlation between the self-reported political orientation and the scores on the Protection subscale.

Next, we explored whether Nurturance and Protection subscales as defined by Hofer et al. (2018) were differentially associated with political orientation. To this end, a regression analysis on political orientation was carried out including the scores on the Nurturance and Protection subscales as predictors while also controlling for participants’ age, gender, and parenthood status. Gender was a significant predictor, *β* = −0.106, *p* = 0.02, 95% CI [−10.79, −0.914], whereas the effect of participants’ age felt short of significance, *β* = 0.115, *p* = 0.05, 95% CI [0.004, 0.375]. Most importantly, scores on the Protection subscale were strongly associated with political orientation, *β* = 0.202, *p* < 0.001, 95% CI [3.43, 9.09] (see Figure 1), whereas scores on the Nurturance subscale were not, *β* = 0.036, *p* = 0.480, 95% CI [−2.07, 4.40]. This suggests that political orientation might be more related to the motivation to protect children rather than to the motivation to provide them with nurturance.

## General discussion

In the present work we aimed at validating the Italian version of the *Parental Care and Tenderness* scale (PCAT; Buckels et al., 2015) by assessing its psychometric properties and testing theoretical predictions derived from previous work that had relied on such scale (e.g., Hofer et al., 2018; Kerry and Murray, 2018). The Italian version of the PCAT showed a high reliability and its internal structure closely reproduced the one reported by Buckels et al. (2015) with a North American sample. In particular, 5 different factors could be identified corresponding to Caring, Protection, Liking, Tenderness-Positive, and Tenderness-Negative. The analysis of the responses provided by male vs. female respondents, and by parents vs. non-parents, supported the validity of the PCAT. Indeed, as theoretically predicted, female respondents and parents displayed stronger parenting motives. In addition, we tested the validity of the PCAT with a novel approach, namely investigating whether the self-reported parenting motivation is predictive of the spontaneous behavioral responses towards children, as assessed by the VAAST (Aubé et al., 2019). The VAAST enabled to identify the individual differences in the tendency to approach infant vs. adult targets. Results clearly showed that higher scores on the PCAT were associated with a predisposition to more quickly move toward infants. This finding is consistent with previous research evidence indicating that the exposure to infant faces, as compared to adult faces, activates brain circuits which are critical for the preparation of social interaction (e.g., premotor cortex; Caria et al., 2012; see also Sherman et al., 2009). Accordingly, in the present study individuals with stronger parenting motives displayed an increased readiness to approach children. From an evolutionary perspective, it can be assumed that such readiness to approach children has implications for an efficient regulation of the responses to the needs of children, thus being an adaptive response aimed at maximizing the survival of the offspring. More generally, findings indicate that individual differences in the self-reported predisposition to take care of children are not only associated with deliberate behaviors, but also with less controlled behavioral and affective responses (see Senese et al., 2013). Future research should more directly focus on how parenting-related motives affect the various actual behaviors (e.g., physical proximity, smiling) that are displayed in the course of interactions with children, both when there is a direct kinship relationship and when it is not present. This will allow ascertaining whether the self-reported differences in parenting motives and the associated approach tendencies, as assessed through computerized tasks (e.g., the VAAST), do actually allow us to predict the quality of the adult-child interactions. In addition, future research should address a limitation of the present research namely the involvement of a sample that is not necessarily representative of the whole population.

Recently, the study of parental motives has been extended by investigating their link with political attitudes (Kerry and Murray, 2018, 2020; Kerry et al., 2022). The findings from Study 3 further corroborate the idea that higher conservatism is associated with stronger parenting motives. Importantly, this emerged regardless of the actual parenthood status that, in turn, was not a significant predictor of political orientation. Hence, psychological individual differences appeared to play the most relevant role, strongly supporting the conclusion provided by Kerry and Murray (2018, p. 92) that “the psychological predilection towards parenting may be a stronger predictor of conservatism (…) than objective parenthood status.”

### The distinction between nurturance and protection

Recently it has been proposed that parental care motives can be distinguished in two primary distinct motivational factors, namely nurturance and protection (Hofer et al., 2018). Hofer et al. (2018), moving from a reanalysis of the data from Buckels et al. (2015), provided evidence that nurturance and protection were indeed the two major conceptual factors underlying the 25-item PCAT scale. Notably, the two identified factors were found to be unique predictors of different attitudes and judgments (Hofer et al., 2018). Following the same analytical strategy, we here obtained support for the presence of the two aforementioned conceptual factors. As in Hofer et al. (2018), the scores on the nurturance and protection subscales were moderately correlated with each other, and they were unique predictors of relevant outcomes. Indeed, the spontaneous behavioral tendencies triggered by the exposure to infant (vs. adult) targets were associated with the individual differences in parental nurturance, but not in parental protection. Respondents with higher scores on the Nurturance subscale were relatively faster in approaching images displaying infants, suggesting that the self-reported motivation to interact and fulfill the psychological and practical needs of children is significantly associated with a spontaneous tendency to move towards children. In contrast, individual differences in parental protection, but not in parental nurturance, emerged to be associated with a more conservative political orientation. This finding is consistent with the observation that conservative individuals often appear to be more sensitive to threats as compared to liberals (e.g., Carraro et al., 2011; Hibbing et al., 2014) especially in the case of physical threats (Crawford, 2017). Conservative attitudes may be conceived as a functional response to perceived threats (Jost et al., 2003) and people who perceive the world as a dangerous place replete with physical threats tend to embrace more conservative political views (Duckitt, 2001). Accordingly, the strength of the reactions to the situations of potential physical harm to children were found to be strongly related to political orientation, even after accounting for the impact of other relevant social variables.

### Conclusion

The present work demonstrated that the Italian version of the PCAT displays very good psychometric properties and an internal structure that closely reproduces the one obtained with North American samples (Buckels et al., 2015). Further evidence was also provided about the validity of the scale by showing its predictive value with respect to spontaneous behavioral tendencies. Finally, findings indicated the importance of the conceptual distinction between two key parental motives (i.e., nurturance and protection) and their unique role in the prediction of relevant phenomena.

## Data availability statement

The datasets presented in this study can be found in online repositories. The names of the repository/repositories and accession number(s) can be found at: https://osf.io/59bf6/files/osfstorage.

## Ethics statement

The studies involving human participants were reviewed and approved by Psychology Ethics Committee of the University of Padova. The patients/participants provided their written informed consent to participate in this study.

## Author contributions

LCas, TG, and LCar: contributed to conception and design of the study. TG: organized the database. TG and LCar: performed the statistical analyses. LCas: wrote the first draft of the manuscript. All authors contributed to manuscript revision, read, and approved the submitted version.

## Conflict of interest

The authors declare that the research was conducted in the absence of any commercial or financial relationships that could be construed as a potential conflict of interest.

## Publisher’s note

All claims expressed in this article are solely those of the authors and do not necessarily represent those of their affiliated organizations, or those of the publisher, the editors and the reviewers. Any product that may be evaluated in this article, or claim that may be made by its manufacturer, is not guaranteed or endorsed by the publisher.
